# Circulating microRNAs in Patients with Shiga-Toxin-Producing *E. coli* O104:H4 Induced Hemolytic Uremic Syndrome

**DOI:** 10.1371/journal.pone.0047215

**Published:** 2012-10-11

**Authors:** Johan M. Lorenzen, Jan Menne, Bernhard MW. Schmidt, Mascha Schmidt, Filippo Martino, Robert Dietrich, Senguel Samiri, Hans Worthmann, Meike Heeren, Karin Weissenborn, Hermann Haller, Mario Schiffer, Jan T. Kielstein, Thomas Thum

**Affiliations:** 1 Institute of Molecular and Translational Therapeutic Strategies (IMTTS), Hannover, Germany; 2 Division of Nephrology and Hypertension, Department of Medicine, Hanover Medical School, Hannover, Germany; 3 Department of Neurology, Hanover Medical School, Hannover, Germany; 4 Centre for Clinical and Basic Research, IRCCS San Raffaele, Rome, Italy; Universität Würzburg, Germany

## Abstract

**Background:**

In early May 2011, an outbreak of hemorrhagic colitis associated with hemolytic–uremic syndrome (HUS) first developed in Northern Germany and spread to 15 other countries in Europe. The outbreak-strain O104:H4, which combined virulence factors of typical enteroaggregative and Shiga-Toxin–producing *E. coli* was associated with an unusual high rate of hemolytic uremic syndrome. Also an unexpected high rate of coma and seizures leading to mechanical ventilation and ICU treatment was observed. MicroRNAs are small ribonucleotides orchestrating gene expression. We tested whether circulating microRNAs in serum of HUS patients during the 2011 epidemics are altered in this patient cohort and related to clinical manifestations.

**Methodology/Principal Findings:**

We profiled microRNAs using RNA isolated from serum of patients and healthy age-matched controls. The results were validated in 38 patients at baseline, 29 patients during follow-up and 21 age-matched healthy controls by miRNA-specific quantitative RT-PCR. Circulating levels of miR-24, miR-126 were increased in HUS patients versus controls. There was no association between these microRNAs and renal function or the need for renal replacement therapy. In contrast, levels of miR-126 were associated with neurological symptoms at baseline and during follow-up. In addition, miR-126 (on admission) and miR-24 (on admission and during follow-up) were associated with platelet count.

**Conclusions/Significance:**

Circulating microRNAs are strongly altered in this patient cohort and associated with neurological symptoms as well as platelet count.

## Introduction

In early May 2011, an outbreak of hemorrhagic colitis associated with hemolytic–uremic syndrome (HUS) developed in Northern Germany but soon spread to 15 other countries in Europe. At the end of this outbreak more than 800 cases of HUS (32 deaths) had been reported to the Robert Koch Institute, Germany`s national center for communicable disease prevention and control [1]. One remarkable difference to prior outbreaks was the frequency of severe neurological complications ranging from headaches, confusion, speech abnormalities, to personality changes, impaired vision, and seizures. The diagnosis of HUS is fulfilled if at least two out of three following findings are present: thrombocytopenia (platelet count of <150×10^9^/l), microangiopathic hemolytic anemia (LDH >250 U/l and hemoglobin <13.5 g/dl), and acute kidney injury [2]. Typical hemolytic uremic syndrome caused by enterohemorrhagic Escherichia coli (EHEC) follows a unique pathogenesis [2]. Following adhesion to the endothelial cell surface Shiga toxin, the most important virulence factor of EHEC, is internalized by receptor-mediated endocytosis. Inside the host cell, it inhibits protein synthesis through interaction with the 60S ribosomal subunit, causing cell death [13] [2]. Platelets subsequently attach to the injured endothelium and are thus removed from the circulation. Multiple microthrombi lead to thrombocytpenia. Fragmentation of red blood cells is assumed to result from mechanical breakdown in occluded vessels [2].

**Table 1:** Demographic, clinical and laboratory characteristics of patients.

	Baseline	Follow-up	p-value
Number of patients	38	29	
Sex			
Male (n; %)	10 (26)	4 (14)	n.s.
Female (n; %)			
Age (years)	46 (18–75)	44 (18–75)	n.s.
eGFR (ml/min/kg)	26 (15–50)	15 (10–22)	<0.01
LDH	1022 (790–1460)	920 (595–1221)	n.s.
Platelets (per 1000/mm^3^)	46 (32–64)	62 (35–90)	n.s.
Hb (g/dL)	11 (9.8–12.3)	8 (7.6–9.1)	<0.0001
PE (n)	33	29	0.04
Dialysis (n)	25	26	0.02
Eculizumab (n)	20	20	n.s.
ICU (n)	21	16	n.s.
Neurology (n)			
cerebellar symptoms	25	19	n.s.
loss of consciousness	11	12	n.s.
Focal- neurological	19	18	n.s.
Psychiatric	22	18	n.s.
Neuro-psychological	26	20	n.s.
Myoclonus	2	2	n.s.
Ictal seizures	9	8	n.s.
miR-24	1.6×10^−3^ (2.9×10^−4^–8.6×10^−2^)	1.6×10^–4^ (5.5×10^−5^–4.8×10^−4^)	0.0001
miR-126	1.8×10^−3^ (2.8×10^−4^ 2×10^−2^)	1.2×10^−4^ (3.9×10^−5^–2.1×10^−4^)	<0.0001

eGFR = estimated glomerular filtration rate; Hb = hemoglobin count; ICU = intensive care unit; LDH = Lactic acid dehydrogenase; PE = plasma exchange; Values of circulating miRNAs represent a ratio between the expression of miRNAs of interest normalized to spiked-in C.elegans control miRNAs.

MiRNAs are endogenous, single-stranded molecules consisting of approximately 22 non-coding nucleotides. A disease-specific role for miRNAs has been shown in various genetic gain and loss of function studies [3]–[4]. Disease initiation might ensue following deregulation of miRNAs through a severe disturbance of downstream gene networks and signaling cascades [5]. However, recent studies demonstrated miRNAs to also be detectable in the circulation and useful as biomarkers for diseases [6]–[9]. MiRNAs are present in the blood in a remarkably stable form that even withstand repetitive freezing/thawing cycles and are protected against RNases [7] [10].

In the present study we tested the hypothesis that circulating microRNAs detected in the circulation of patients presenting with HUS due to an infection with STEC O104:H4 are altered and might serve as early biomarkers for complications during the course of the disease. We therefore analyzed circulating miRNA levels in plasma of 38 HUS patients on admission, 29 patients after the third therapeutic plasma exchange (follow-up) and 21 age-matched healthy controls.

**Table 2:** Genome-wide qRT-PCR-based screen of deregulated miRNAs in HUS patients in comparison to healthy controls.

	2 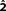 (-Avg.(Delta(Ct))				
	Control Group	HUS	Fold Change	95% CI	Fold Regulation
**Repressed in HUS**					
hsa-miR-184	0.012	0.003	**0.3**	(0.17, 0.40)	−3.5
hsa-miR-375	0.015	0.004	**0.3**	(0.11, 0.47)	−3.4
hsa-miR-206	0.010	0.003	**0.3**	(0.31, 0.36)	−3.0
hsa-miR-96	0.009	0.003	**0.3**	(0.32, 0.38)	−2.9
hsa-miR-200a	0.009	0.003	**0.4**	(0.33, 0.37)	−2.8
hsa-miR-34a	0.009	0.004	**0.4**	(0.30, 0.44)	−2.7
hsa-miR-124	0.011	0.004	**0.4**	(0.19, 0.58)	−2.6
hsa-miR-205	0.010	0.004	**0.4**	(0.23, 0.54)	−2.6
hsa-miR-204	0.009	0.004	**0.4**	(0.31, 0.46)	−2.6
hsa-miR-192	0.096	0.039	**0.4**	(0.25, 0.55)	−2.5
hsa-miR-141	0.009	0.004	**0.4**	(0.27, 0.55)	−2.5
hsa-miR-210	0.009	0.004	**0.4**	(0.34, 0.52)	−2.3
hsa-miR-296-5p	0.011	0.005	**0.4**	(0.24, 0.63)	−2.3
hsa-miR-193a-5p	0.009	0.004	**0.5**	(0.32, 0.58)	−2.2
hsa-miR-150	1.815	0.849	**0.5**	(0.42, 0.52)	−2.1
hsa-miR-133b	0.009	0.005	**0.5**	(0.26, 0.80)	−1.9
hsa-miR-10b	0.010	0.005	**0.5**	(0.36, 0.71)	−1.9
**Induced in HUS**					
hsa-miR-126	4.599	6.704	**1.5**	(0.99, 1.92)	1.5
hsa-miR-191	3.519	5.159	**1.5**	(1.35, 1.58)	1.5
hsa-miR-23a	6.344	9.547	**1.5**	(1.18, 1.83)	1.5
hsa-miR-26a	4.263	6.510	**1.5**	(1.25, 1.80)	1.5
hsa-miR-24	2.085	3.192	**1.5**	(1.35, 1.72)	1.5
hsa-miR-221	0.854	1.350	**1.6**	(1.40, 1.76)	1.6
hsa-miR-30d	2.020	3.206	**1.6**	(1.26, 1.92)	1.6
hsa-miR-15a	0.060	0.099	**1.6**	(1.09, 2.19)	1.6
hsa-miR-30e	1.409	2.406	**1.7**	(1.44, 1.97)	1.7
hsa-miR-27a	1.469	2.635	**1.8**	(1.34, 2.24)	1.8
hsa-miR-103a	0.341	0.774	**2.3**	(1.97, 2.57)	2.3
hsa-miR-143	0.040	0.093	**2.3**	(1.20, 3.42)	2.3

**Figure 1:**
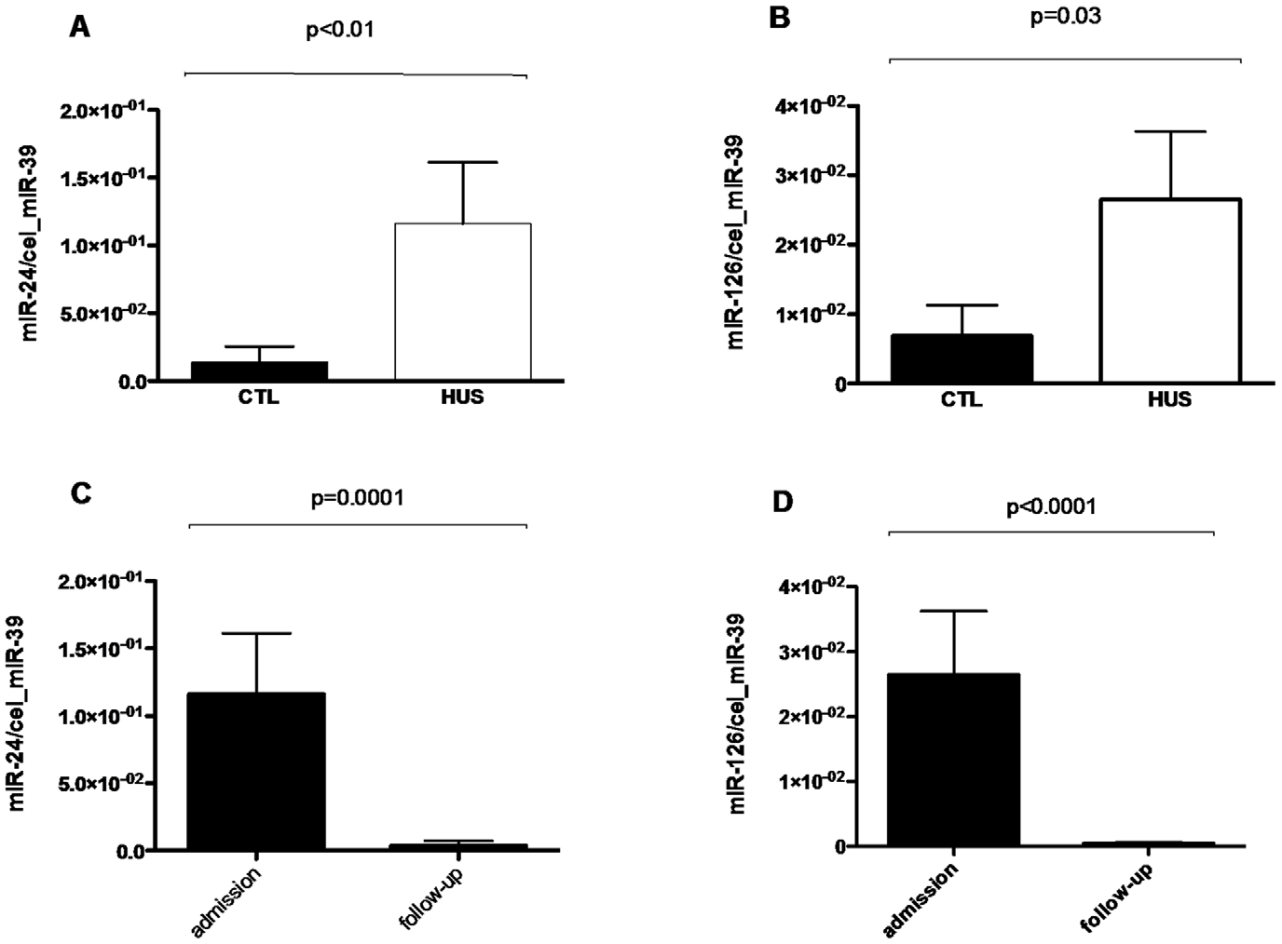
Circulating levels of miR-24 and miR-126 in patients with EHEC-HUS (HUS, n = 38) compared to healthy controls (CTL, n = 21) at baseline (A, B) and during follow-up (n = 29; C, D) are shown. Data are displayed as mean ± standard deviation (SD).

**Figure 2:**
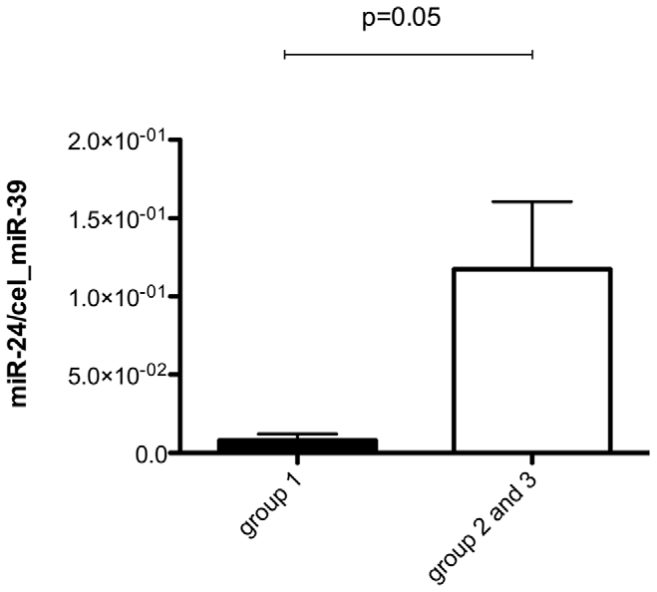
Circulating levels of miR-24 are shown in patients with slight neurological impairment (group 1) compared to patients with moderate and severe impairment (group 2 and 3).

**Figure 3:**
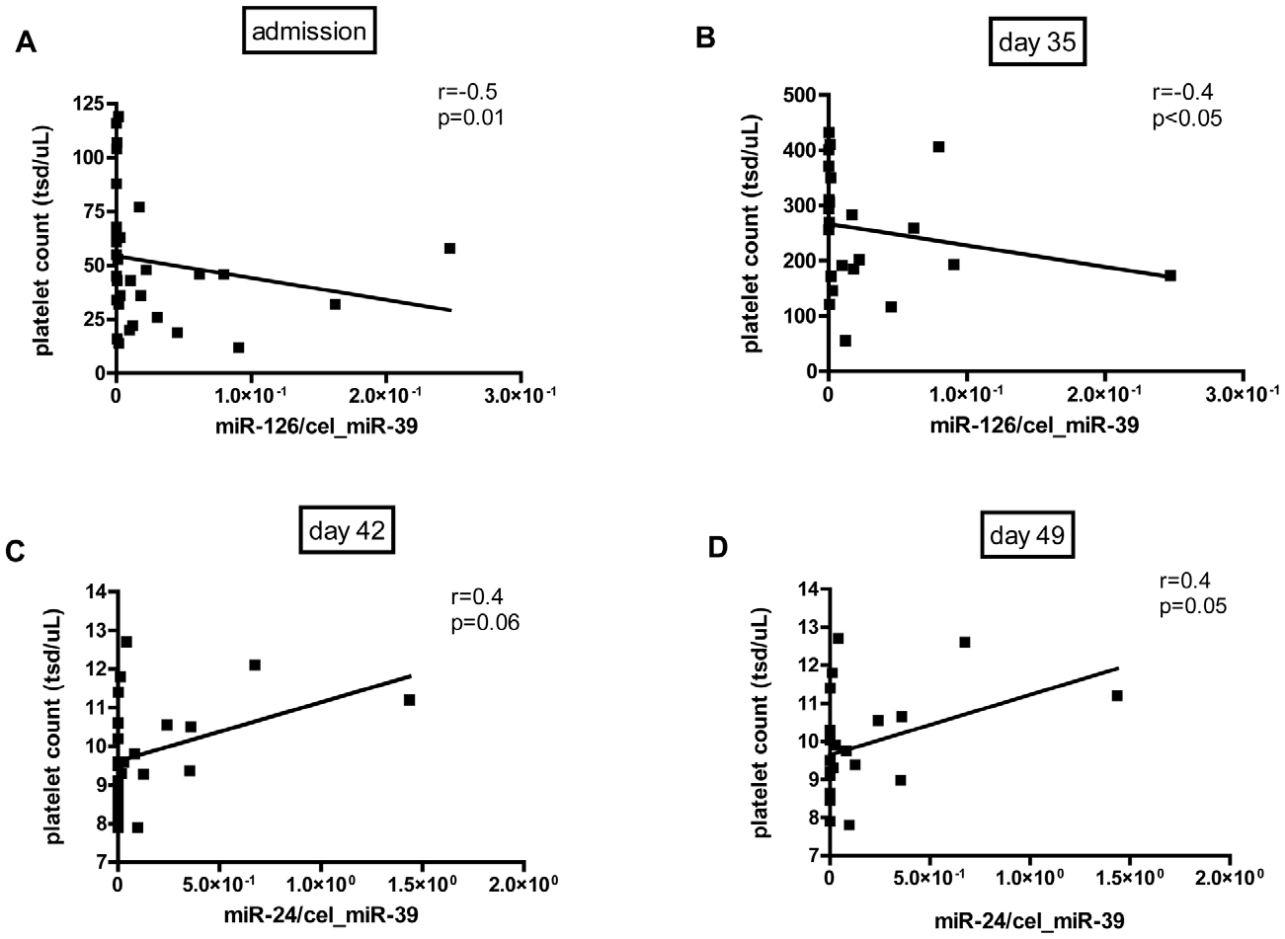
Correlation between miR-126 and platelet count on admission (A) and day 35 (B) as well as correlation between miR-24 and platelet count on day 42 (C) and day 49 (D) is shown.

**Figure 4:**
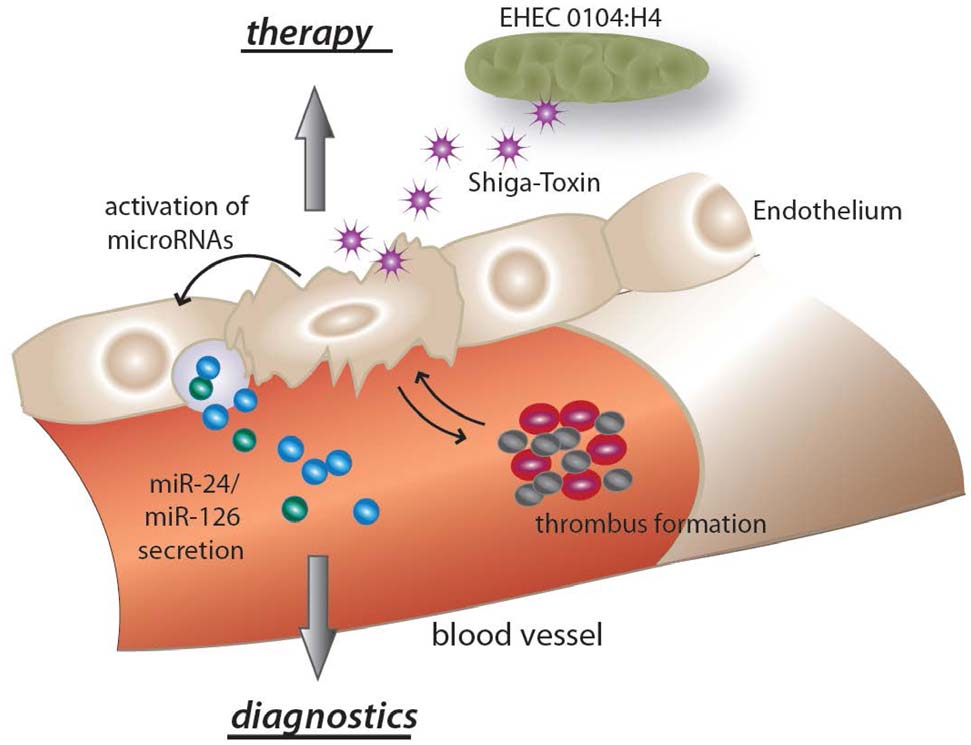
The proposed schematic action of miRNAs in Shiga toxin associated typical HUS is shown. Shiga toxin targets and destroys endothelial cells, thereby inducing the detrimental cascade of platelet consumption, fragmentation of red blood cells and thrombotic microangiopathy. Endothelial apoptosis results in an induction of miR-24 and miR-126. MiR-24 is subsequently released to the circulation and perpetuates the disease process by activating adjacent endothelial cells. MiR-126 in apoptotic bodies sends survival signals to adjacent endothelium.

## Materials and Methods

### Patients and Sample Collection

Thirty-eight patients presenting with EHEC-HUS to Hannover Medical School during the EHEC serotype O104:H4 outbreak in 2011 were included in this study. Written informed consent was obtained from all patients. Treatment consisted of plasma exchange in 33 patients (87%). Severe cases also received the humanized monoclonal antibody eculizumab (n = 20, 53%). Twenty-five patients (66%) required haemodialysis (HD), 21 patients (55%) were admitted to the intensive care unit. A number of patients developed neurological symptoms (see **table 1**). The study protocol was carried out in accordance with the Declaration of Helsinki and approved by the Institutional Review Board at Hannover Medical School.

### Sampling and Quantification of Circulating miRNAs

Serum samples of all patients were obtained on admission as well as after the third plasmapheresis. Twenty-one age-matched healthy volunteers served as controls. RNA was isolated using the MasterPure™ RNA Purification Kit (Epicentre Biotechnologies) according to the manufacturer’s instructions. Fifty microliters of serum were used for RNA isolation. We supplemented the samples with 1 fmol/µL *Caenorhabditis elegans* miR-39 (cel-miR-39) and normalized serum miRNAs to this control as described previously [6], [7].

### miRNome Profile Analysis

To assess an impact of *Escherichia coli* O104:H4-associated HUS on circulating miRNAs, we conducted a global miRNA expression analysis. Pooled total RNA from 10 patients with *Escherichia coli* O104:H4-associated hemolytic uremic syndrome on admission, 10 patients after the third plasmapheresis and 10 age-matched healthy control patients was used. Total RNA was processed as described earlier [5].

We employed the 384-well miScript miRNA qRT-PCR array (Qiagen, Germany) according to the manufacturer’s instructions to screen for deregulated miRNAs. Deregulated miRNAs between healthy controls and patients with HUS are shown in **Table 2**.

### Detection and Quantification of miRNAs by Quantitative PCR

We focused on miR-24 and miR-126 because of the level of deregulation and their proven role in vascular biology [11], [12]. MiR-24 and miR-126 were validated by quantitative miRNA RT-PCR technology (TaqMan MicroRNA Assays, Applied Biosystems, Foster City, California) in 38 HUS patients on admission, 29 patients after the third plasmapheresis and 21 age-matched healthy controls. We used highly target-specific stem loop structure and reverse transcription primer, and after reverse transcription used specific TaqMan hybridization probes for miRNA amplification. This allowed for high specificity for only the mature miRNA and formation of a reverse transcription primer/mature miRNA chimera, extending the 5′ end of the miRNA. Values were normalized to spiked-in cel-miR-39.

### Statistical Analysis

Continuous variables are expressed as medians with corresponding 25^th^ and 75^th^ percentiles (IQR) and are compared by using the Mann-Whitney rank sum test or the Kruskal Wallis one-way analysis of variance. Age is displayed as median (Min – Max). Categorical variables are compared using the Χ^2^ test. Variables are assessed for normal distribution using the Kolmogorov-Smirnov test. Correlations between variables are assessed by the Spearman rank correlation coefficient. All statistical analyses are performed with the SPSS package (SPSS Inc., Chicago, IL, USA) and GraphPad Prism software (GraphPad Prism Software Inc. San Diego, California, USA). Two-sided p-values <0.05 were considered statistically significant for all statistical procedures used.

## Results

### Circulating miRNAs are Severely Altered in Patients with Escherichia Coli O104:H4-Associated HUS

The clinical characteristics of the whole cohort of HUS patients (n = 38) are shown in **Table 1**. The levels of 29 circulating miRNAs differed between patients and healthy controls (**Table 2**). Due to their level of deregulation and described role in endothelial biology we investigated the levels of circulating miR-24 and miR-126 in a validation set of 38 patients on admission, 29 patients after the third therapeutic plasma exchange and 21 age-matched controls by using TaqMan qPCR. As shown in **Figure 1a, b**, the results of the miRNA profile could be confirmed in the entire cohort identifying miR-24 and miR-126 to be up-regulated (p<0.01 and p = 0.03, respectively) in patients with HUS (n = 38) compared to healthy controls (n = 21) at baseline. Levels of these miRNAs were significantly reduced by therapeutic plasma exchange (all p<0.01) (see **Figure 1c, d**). Our study population consisted of 28 females (74%) and 10 males (26%). None of the miRs differed between male and female patients (miR-24: p = 0.5; miR-126: p = 0.4). The median age of patients was 46 years (18–82). Healthy controls consisted of 14 female and 7 male patients (median age: 47 years (18–82)). MiR-126 (r = -0.4, p = 0.04) negatively correlated with age, while miR-24 did not show an association. A difference concerning circulating levels of the investigated miRNAs could not be observed in patients treated with the monoclonal antibody eculizumab (miR-24: p = 0.2; miR-126: p = 0.9).

### Circulating miRNAs and Kidney Function

Levels of miR-24 (r = 0.6, p = 0.007) after the third therapeutic plasma exchange (n = 29) correlated with estimated glomerular filtration rate (eGFR) on day 42. We did not detect an association between individual levels of circulating miRs on admission (n = 38) and the need for renal replacement therapy (miR-24: p = 0.8; miR-126: p = 0.8).

### Circulating miRNAs and Neurological Symptoms

At baseline (n = 26) and during follow-up (n = 20) a number of patients developed severe neurological deficits. On admission levels of miR-24 were lower in patients with slight neurological impairment (group 1) compared to moderate and severe impairment (group 2 and 3) (p = 0.05, see **Figure 2**). Moreover, levels of miR-126 on admission showed borderline correlation with the overall neurological score on day of diagnosis (r = 0.3, p = 0.08).

### Platelet Count and Circulating miRNAs

Thrombocytopenia is a major hallmark of HUS. Levels of miR-126 negatively correlated with platelet count on admission (n = 38) (r = -0.5, p = 0.01, Figure 3A) and on day 35 of hospital stay (n = 29) (r = -0.4, p<0.05, Figure 3B), while levels of miR-24 were positively associated with platelet count on day 42 (r = 0.4, p = 0.06, Figure 3C) and day 49 (r = 0.4, p = 0.05, Figure 3D).

## Discussion

Our study is the first clinical evaluation of circulating miRNAs in patients presenting with hemolytic uremic syndrome due to an infection with Shiga toxin-producing enterohemorrhagic Escherichia coli O104:H4. The results are as follows: (1) HUS leads to a severe deregulation within the circulating miRNA pool, (2) levels of endothelial-enriched miR-24 and miR-126 increased in this patient cohort, (3) miR-126 was associated with onset of neurological symptoms. (4) MiR-24 and miR-126 are associated with platelet count during HUS.

We provide proof of the successful detection of circulating microRNAs in patients with HUS. In the past few years, research efforts aimed towards the identification of pathogenetic factors during the development of typical HUS. The underlying mechanistic basis for deregulated gene expression during an HUS remains an area of utmost interest, which has not been completely solved. MiRNAs regulate a large fraction of the genome and might therefore be involved in this process.

The results of our study show that specific microRNAs might be involved in the unique pathogenetic process of endothelial injury during HUS.

MiR-126 was shown to be released from endothelial apoptotic bodies and to induce the release of CXCL12 to counteract further endothelial cell apoptosis [12]. In addition, miR-126 promoted the incorporation of Sca-1^+^ progenitor cells to the injured endothelium [12]. We found miR-126 to be up-regulated in patients with HUS. It is conceivable, that miR-126 is released from apoptotic endothelial cells, which have been targeted by circulating shiga toxin during the induction of HUS. In HUS miR-126 might also provide survival signals to adjacent endothelial cells to limit the disease process. Our findings concerning miR-24 are in line with this hypothesis. MiR-24 is enriched in endothelial cells and highly up-regulated during cardiac ischemia [11]. Furthermore, overexpression of miR-24 induces endothelial cell apoptosis, abolishes endothelial capillary network formation and inhibits cell sprouting from endothelial cells in vitro [11]. Circulating levels of miR-24 are highly increased in patients with HUS, possibly reflecting endothelial damage and further availing the induction of endothelial cell apoptosis. We propose miR-126 and miR-24 to represent two ends of a detrimental spectrum in typical HUS. The proposed scheme of miRNA action in HUS is depicted in **Figure 4** **.**

Treatment of severe HUS is comprised of immediate plasma exchange to reverses the platelet consumption responsible for the thrombus formation and symptoms [14]. Plasma exchange in our cohort lowered elevated levels of endothelial-enriched microRNAs.

Recently, the humanized monoclonal antibody eculizumab was evaluated for the first time in three pediatric patients with EHEC-HUS and shown to successfully reverse severe neurological complications of HUS [15] after an initial report of its successful administration in atypical HUS, which is thought to result from complement-mediated endothelial damage and platelet activation [16] [17], [18]. Patients with EHEC-associated HUS were shown to present with elevated levels of the complement factors Bb and sC5b-9 at presentation [19], indicating that activation of the alternative complement pathway might play a role in the pathogenesis of EHEC-associated HUS.

Eculizumab, initially approved for the treatment of paroxysmal nocturnal hemoglobinuria (PNH) [20], binds to the complement protein C5, thereby inhibiting its cleavage to C5a and C5b. This prevents the generation of the terminal complement complex C5b–C9 [21]. Prospective studies on the use of eculizumab in EHEC-HUS patients of the German epidemic of 2011 are currently ongoing and under evaluation. However, in our study we did not find an association between individual levels of miRNAs implicated in endothelial biology and eculizumab treatment, possibly pointing to the lack of an effect of eculizumab on the endothelium.

Our data suggest that circulating miRNAs might be an interesting readout for therapeutic interventions. Interfering with miRNAs in other organ systems has already been shown to alter pathophysiological processes [22].

There are certain limitations to our study: the expression of the enzyme dicer, which is critical for miRNA biogenesis, has been shown to be influenced by cellular stressors [23]. Thus, dysregulation of miRNAs in our cohort might also be a reflection of an impairment of the cellular miRNA processing machinery. Secondly, our study is merely descriptive in nature. We are unable to provide molecular insights into the underlying mechanisms of miRNA dysregulation in HUS. Clearly, further experimental studies are critical to elucidate the underlying mechanisms.

Currently, a stable circulating microRNA is not available, thereby impeding the possibility to use certain microRNAs as normalizing controls. Thus, we did not normalize levels of circulating miRNAs to a “housekeeping” microRNA. Instead, we supplemented the serum samples with recombinant *C. elegans* miR-39, which can be specifically detected by TaqMan-qRT-PCR, to normalize potential differences in the efficiency of RNA isolation as shown previously [6], [7]. Due to the profound impact of various factors (e.g. hypoxia, toxins) it is unlikely (dependent upon the disease condition investigated) that a single microRNA might serve as a stable control microRNA.

In conclusion, the present study provides first insights into circulating levels of miRNAs in patients with Escherichia coli O104:H4-associated hemolytic uremic syndrome. MRNAs can be reliably detected in this patient cohort. We identified a unique profile of deregulated miRNAs, which might be involved in endothelial damage and response to apoptosis thereby influencing the disease process.
